# Cross-cultural adaptation and psychometric validation of the Chinese version of the Professional Interpersonal Competency Assessment Scale for novice nurses

**DOI:** 10.3389/fpubh.2026.1806827

**Published:** 2026-08-07

**Authors:** Qi Zhang, Yuxia Chang, Xu Han, Weihua Liu, Yanwei Cheng, Jiange Zhang

**Affiliations:** 1 Department of Emergency, Henan Provincial People’s Hospital, People’s Hospital of Zhengzhou University, People’s Hospital of Henan University, Zhengzhou, China; 2 Henan Provincial Key Medicine Laboratory of Nursing, Henan Provincial People’s Hospital, Zhengzhou, China; 3 Nursing Department of Henan Provincial People’s Hospital, People’s Hospital of Zhengzhou University, People’s Hospital of Henan University, Zhengzhou, China

**Keywords:** novice nurses, perception, professional interpersonal competency, psychometric properties, reliability, validity

## Abstract

**Background:**

Professional interpersonal competency is crucial for mitigating turnover and enhancing practice effectiveness among novice nurses. However, a specific instrument for assessing this competency in the Chinese context is currently lacking.

**Objective:**

This study aimed to translate the Professional Interpersonal Competency Assessment Scale for Novice Nurses (PICASN) into Chinese and evaluate its psychometric properties.

**Design:**

A cross-sectional, quantitative design was employed.

**Methods:**

Following Brislin’s translation model, the PICASN underwent translation, back-translation, synthesis, cultural adaptation, and pilot testing to create the Chinese version (C-PICASN). Between August and October 2025, novice nurses were recruited via convenience sampling from tertiary grade-A hospitals in Zhengzhou, Henan Province, to assess the scale’s reliability and validity.

**Results:**

The final C-PICASN consists of 27 items across two factors. Exploratory factor analysis extracted two factors with eigenvalues >1, accounting for 68.140% of the total variance. Confirmatory factor analysis demonstrated acceptable model fit (χ^2^/df = 4.038, RMSEA = 0.080, SRMR = 0.040, CFI = 0.925, TLI = 0.916, IFI = 0.925). Content validity indices were satisfactory (I-CVI = 0.875–1.000, S-CVI/Ave = 0.991). The scale exhibited excellent internal consistency (Cronbach’s *α* = 0.975), test–retest reliability (ICC = 0.909), and split-half reliability (0.952).

**Conclusion:**

The Chinese version of the PICASN demonstrates good reliability and validity, supporting its use as a reliable tool for assessing professional interpersonal competency among novice nurses in China.

## 1 Introduction

Early-career attrition remains a persistent workforce challenge in nursing, and workplace relationship difficulties are repeatedly identified as proximal drivers of novice nurses’ turnover intention and resignation (1, 2). During the transition from student to independent practitioner, novice nurses must rapidly adapt to new roles, expectations, and team norms, and the quality of socialization and transitional support is closely linked to early adjustment and retention (3, 4). Mentoring and perceived support have likewise been emphasized as actionable strategies to strengthen novice nurses’ integration and reduce early departure (5).

Professional interpersonal competence is fundamental to nursing practice because it underpins therapeutic nurse–patient relationships, collaboration with families, and effective teamwork across disciplines, thereby influencing care quality and safety (4). Patient-centered care frameworks further highlight the importance of respectful communication and relational continuity in shaping patients’ experiences and trust (6). Within healthcare teams, hierarchical structures and power gradients can inhibit junior staff from speaking up, which may undermine teamwork and patient safety (7). In cultural contexts that strongly emphasize seniority and professional status, hierarchy may further constrain assertive communication, with particular relevance to East Asian nursing environments (8, 9). At the individual level, professional values, self-efficacy, humility, and self-care capacities have also been linked to nurses’ performance, collaboration, and well-being, reinforcing the need to understand and support interpersonal competence during early career development (9). Broader scholarship on soft skills (10), digital-era competencies (11), values-based leadership (12), and supportive relationships (13) similarly underscores interpersonal competence as a core professional capability in contemporary nursing practice.

Despite its relevance, measurement of interpersonal competence has often targeted students or other professional groups. Although instruments have been developed and validated for nursing students (14), public health midwives (15), and healthcare professionals (16), these tools may not fully capture the interpersonal demands encountered by novice nurses during early professional adjustment. To address this gap, Sato and colleagues (17) developed the Professional Interpersonal Competency Assessment Scale for Novice Nurses (PICASN). The scale comprises two domains: Basic Competencies as a Novice Nurse and Relationship-Building Skills within the Healthcare Team, and has demonstrated acceptable reliability and validity among Japanese novice nurses (17).

For use in a new language and cultural setting, instruments require rigorous translation and psychometric validation to ensure linguistic accuracy and conceptual equivalence and to minimize construct drift. Methodological guidance recommends transparent reporting (including for observational designs), adequate sample sizes, and factor-analytic evaluation of dimensionality and model fit, together with evidence for reliability, content validity, and construct validity. Recent Chinese adaptations and validations of health-related instruments support the feasibility and value of such workflows in producing culturally appropriate measures. Accordingly, this study aimed to translate and culturally adapt the PICASN into Chinese and to evaluate the psychometric properties of the Chinese version (C-PICASN) among novice nurses in China, including item performance, content validity, construct validity (exploratory and confirmatory factor analyses), internal consistency, split-half reliability, and test–retest reliability.

## 2 Methods

### 2.1 Study design and reporting

**Figure 1:**
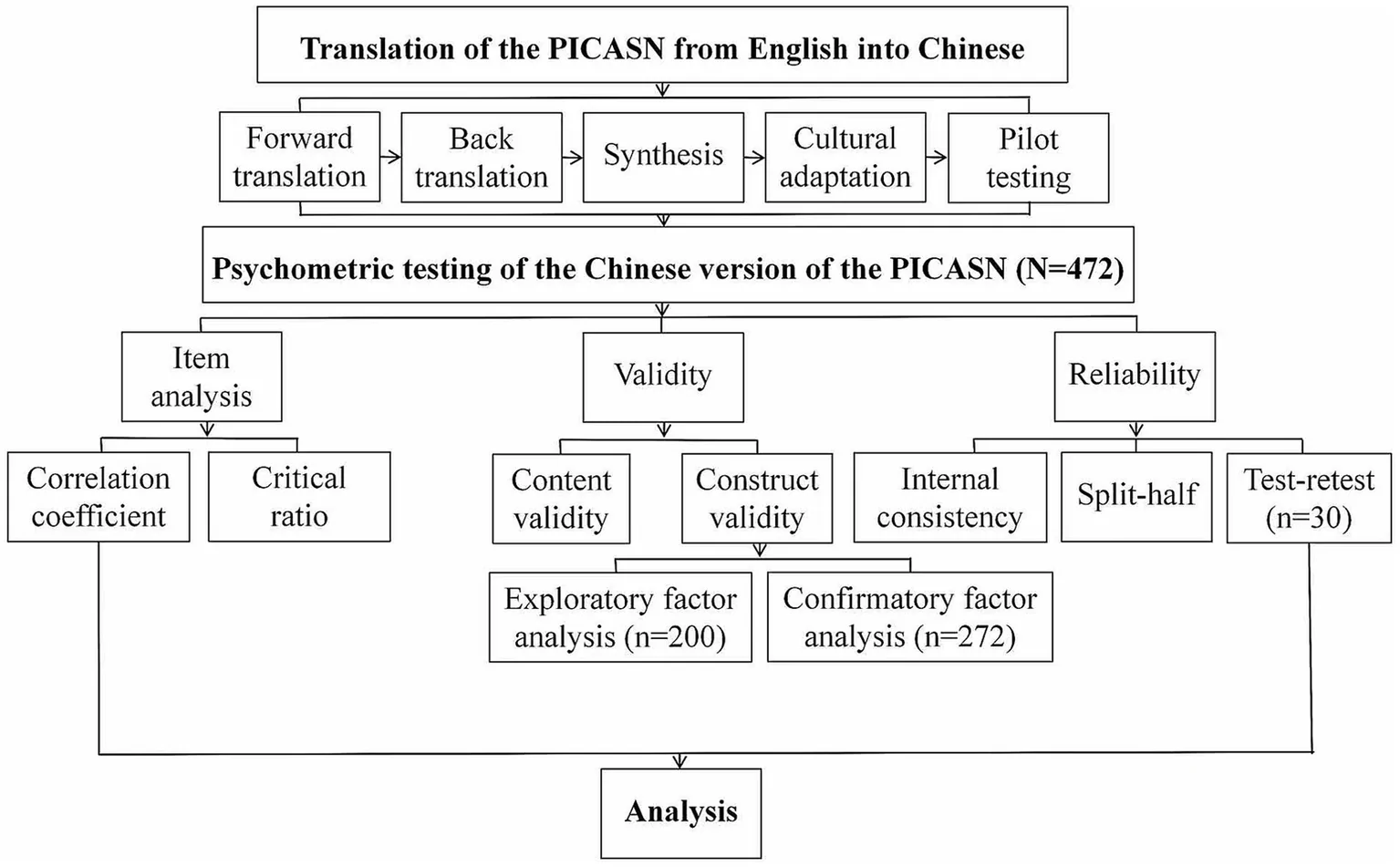
Translation and psychometric flow chart of the C-PICASN.

This cross-sectional quantitative study adhered to the Strengthening the Reporting of Observational Studies in Epidemiology (STROBE) guidelines for reporting observational studies and instrument validation (18, 19). The primary aim was to validate the psychometric properties of the Chinese version of the Professional Interpersonal Competency Assessment Scale for Novice Nurses (C-PICASN). The study comprised two phases: (i) translation and cross-cultural adaptation and (ii) psychometric validation (Figure 1). The study protocol was reviewed and approved by the institutional ethics committee, and written informed consent was obtained from all participants prior to data collection.

### 2.2 Ethical considerations

The study protocol was approved by the Ethics Committee of Henan Provincial People’s Hospital and conducted in accordance with the Declaration of Helsinki. Prior to participation, all participants received detailed information regarding the study purpose, procedures, and potential benefits. Written informed consent was obtained from each participant. Participation was voluntary, and participants could withdraw at any time without consequences. Data were collected anonymously and handled confidentially for research purposes only.

### 2.3 Participants and setting

From August to October 2025, a cross-sectional study was conducted in Henan Province, China, to examine the psychometric properties of the C-PICASN. Participants were recruited via convenience sampling. Inclusion criteria were: (i) holding a valid nursing practice license; (ii) having 6 months to 5 years of clinical experience; and (iii) providing written informed consent. Exclusion criteria were: (i) interns or nurses in standardized or advanced training programs and (ii) nurses on personal, maternity, or medical leave.

For factor analysis, a sample size of 10–20 times the number of items is recommended (20), with exploratory factor analysis (EFA) requiring >100 participants and confirmatory factor analysis (CFA) > 200 participants (21). Allowing for a 10% invalid response rate (22) the target sample size was calculated as (10–20) × 27 ÷ (1–0.10) ≈ 300–600.

### 2.4 Study procedure

#### 2.4.1 Translation and back-translation of the C-PICASN

After obtaining authorization from the original author, we followed Brislin’s translation and back-translation model (23). (i) Forward translation: Two bilingual translators (one clinical nurse and one English teacher without a medical background) independently produced Chinese versions A1 and A2. The research team reconciled discrepancies to create a unified version (A3). (ii) Back-translation: Two translators with expertise in nursing/psychometrics and overseas medical study experience independently back-translated A3 into English (versions B1 and B2). (iii) Synthesis: Two nursing PhDs compared B1, B2, and the original scale to produce a consolidated back-translated version (B3). The research team then adjusted items for cultural and linguistic appropriateness, resulting in the preliminary Chinese version (C1).

#### 2.4.2 Cultural adaptation of the C-PICASN

Eight experts in clinical nursing, nursing education, and scale development were invited to review the questionnaire. Expert selection criteria included: (1) an associate senior title or higher; (2) a bachelor’s degree or above; and (3) > 10 years of clinical nursing experience. Each expert received a package containing the C1 version, the original English scale, a description of the study purpose, and a 4-point Likert scale evaluation form (1 = not relevant to 4 = highly relevant) to assess language clarity, content relevance, and cultural applicability (22). Experts were given two weeks to complete the evaluation and were encouraged to provide written comments on each item, particularly regarding wording ambiguity, cultural inappropriateness, or conceptual misfit. Following the expert review, we calculated item-level content validity indices (I-CVI) for each item. Items with I-CVI below 0.78 were flagged for revision-. The research team then convened a meeting to review all expert comments and statistical results. Each flagged item was discussed collectively, and revisions were made when at least five of the eight experts (62.5%) identified the same issue or when the I-CVI fell below the threshold. Representative item modifications included the following: Item 18 (original): “It is important for novice nurses to not become emotionally attached to the points raised by nurses in their team.” Experts noted that the phrasing “emotionally attached” was culturally ambiguous in the Chinese context, where emotional restraint in professional settings is valued differently than in Western cultures. The item was revised to: “Novice nurses should avoid excessive empathy that may affect professional judgment and maintain a rational and objective stance when responding to questions from team members,” which better aligns with local nursing perspectives on professional boundaries. These adjustments, informed by both quantitative (I-CVI) and qualitative (expert comments) evidence, yielded the culturally adapted version (C2).

#### 2.4.3 Pilot testing of the C-PICASN

A convenience sample of 40 novice nurses completed the pilot test and provided feedback on completion time, instruction clarity, item comprehension, and ambiguity (22). Ambiguous or poorly worded items were revised accordingly, resulting in the final Chinese version (C3).

### 2.5 Measures

#### 2.5.1 General information questionnaire

Demographic and work-related data collected included gender, age, education, professional title, position, clinical unit, hospital level, years of experience, employment form, specialist nurse certification (national/provincial), prior training in interpersonal communication, experience of being reprimanded by a head nurse, ward atmosphere, and intention to continue working at the hospital.

#### 2.5.2 PICASN

The original Professional Interpersonal Competency Assessment Scale for Novice Nurses (PICASN), developed by Sato et al. (17), assesses professional interpersonal competency through two factors: Basic Competencies as a Novice Nurse (15 items) and Relationship-Building Skills within the Healthcare Team (12 items), totaling 27 items. Items are rated on a 5-point Likert scale from “not at all important” (1) to “very important” (5). Total scores range from 27 to 135, and higher scores indicate greater perceived competency.

### 2.6 Data analysis

Data were analyzed using SPSS 25.0 and AMOS 24.0. Categorical data are presented as frequencies and percentages. Normally distributed continuous data are presented as mean ± standard deviation. Statistical significance was set at *p* < 0.05.

### 2.7 Item analysis

Item analysis employed the correlation-coefficient method and the critical-ratio (CR) method. Participants were ranked by total score, with the top and bottom 27% forming the high- and low-score groups, respectively. Independent-samples t-tests compared item means between groups. Items were considered for deletion if: (i) the score difference between extreme groups was non-significant (*p* > 0.05) or (ii) the item–total correlation was non-significant (p > 0.05) or the correlation coefficient was <0.30 (24).

### 2.8 Reliability

Reliability was assessed via internal consistency (Cronbach’s *α*), split-half reliability, and test–retest reliability. Cronbach’s α > 0.70 indicated acceptable internal consistency (25). Split-half reliability was calculated by correlating scores from odd- and even-numbered item halves, and a coefficient > 0.60 was considered acceptable (26). Test–retest reliability was evaluated using the intraclass correlation coefficient (ICC) in a subsample of 30 nurses who completed the scale again after a two-week interval. An ICC > 0.70 indicated good temporal stability (27).

### 2.9 Validity

Validity was examined through content validity and construct validity. For content validity, eight experts rated each item on the 4-point Likert scale. The item-level content validity index (I-CVI) and scale-level content validity index/average (S-CVI/Ave) were calculated; I-CVI ≥ 0.78 and S-CVI/Ave ≥ 0.80 indicated good content validity (28, 29). For construct validity, the sample was randomly split into Subsamples A and B. Subsample A underwent exploratory factor analysis (EFA) using principal component analysis with varimax rotation. EFA suitability was confirmed by a Kaiser–Meyer–Olkin (KMO) measure > 0.80 and a significant Bartlett’s test of sphericity (*p* < 0.001) (30). In the exploratory factor analysis (EFA), we employed multiple criteria to determine the number of factors to retain (31). First, we examined the scree plot for the point of inflection. Second, we applied the eigenvalue-greater-than-one criterion as an initial screening tool. Third, we conducted parallel analysis which suggested a two-factor solution, consistent with the eigenvalue criterion. Fourth, we required that the retained factors collectively explain at least 50% of the total variance, a commonly recommended threshold for adequate construct representation. Finally, we ensured that each retained factor contained at least three items with primary loadings > 0.40. Subsample B underwent confirmatory factor analysis (CFA) using maximum likelihood estimation. Model fit was assessed using χ^2^/df, root mean square error of approximation (RMSEA), standardized root mean square residual (SRMR), comparative fit index (CFI), Tucker–Lewis index (TLI), and incremental fit index (IFI) (32).

## 3 Results

### 3.1 Participant characteristics

A total of 485 questionnaires were distributed and 472 valid questionnaires were returned, yielding an effective response rate of 97.2% (Table 1). The mean age was 23.5 ± 1.89 years, with 52.3% aged 23–24 years and 84.1% being female. Most participants held a bachelor’s degree (68.4%), while 15.7% had a junior college education or below and 1.9% had a master’s/PhD degree. Regarding professional title, 46.8% were senior nurses, 41.1% were nurses, and 12.1% were supervisor nurses. Participants were mainly from internal medicine (51.5%), followed by intensive care settings including the emergency room, ICU, and operating room (31.8%), surgery (13.1%), and pediatrics, obstetrics, and gynecology (3.6%). More than half worked in tertiary A-level hospitals (59.5%). Most reported contract-based employment (67.8%), while 30.3% were employed through personnel agencies and 1.9% held permanent positions. Specialist nurse certification was reported by 17.8% at the national level and 19.9% at the provincial level. Only 18.9% had received formal professional interpersonal skills training, and 25.8% reported having been scolded by a senior nurse. Most perceived the ward atmosphere as good (93.4%) and intended to continue working at their current hospital (95.6%).

**Table 1:** Demographic and work-related characteristics of the novice nurses (*n* = 472).

Variable		*n*	%
Age(Mean ± SD) 23.5 ± 1.89 years			
	21 ~ 22 years old	126	26.7
	23 ~ 24 years old	247	52.3
	25 ~ 29 years old	85	18.0
	Over 30 years old	14	3.0
Gender			
	Male	75	15.9
	Female	397	84.1
Educational level			
	Junior college and below	74	15.7
	Bachelor’s degree	323	68.4
	Master’s or PhD degree	9	1.9
Professional title			
	Nurse	194	41.1
	Senior nurse	221	46.8
	Supervisor nurse	57	12.1
Department			
	Department of Internal Medicine	243	51.5
	Department of Surgery	62	13.1
	Intensive care (Emergency Room, Intensive Care Unit, Operating Room)	150	31.8
	Pediatrics, Obstetrics and Gynecology	17	3.6
Hospital level			
	Tertiary A-level hospital	281	59.5
	Tertiary B-level hospital	108	22.9
	Secondary A-level hospital	80	17.0
	Secondary B-level hospital	3	0.6
Employment form			
	Contract-based employment	320	67.8
	Personnel agency employment	143	30.3
	Permanent staff employment	9	1.9
Having a Chinese nursing association specialist nurse certificate			
	Yes	84	17.8
	No	388	82.2
Having a provincial specialist nurse certificate			
	Yes	94	19.9
	No	378	80.1
Have you ever attended any professional interpersonal skills training?			
	Yes	89	18.9
	No	383	81.1
Have you ever been scolded by a senior nurse?			
	Yes	122	25.8
	No	350	74.2
Do you feel that the atmosphere in your department is good?			
	Yes	441	93.4
	No	31	6.6
Would you like to continue working at your current hospital?			
	Yes	451	95.6
	No	21	4.4

### 3.2 Item analysis and reliability

As shown in Table 2, item–total correlations for the C-PICASN were all statistically significant (*p* < 0.01) and exceeded the prespecified criterion of 0.30, indicating satisfactory item discrimination. For Factor 1 (Basic Competencies as a Novice Nurse), item–total correlations ranged from 0.660 (Item 2) to 0.873 (Item 12). For Factor 2 (Relationship-Building Skills within the Healthcare Team), item–total correlations ranged from 0.569 (Item 24) to 0.878 (Item 16). No items met the pre-specified deletion criteria, therefore, all 27 items were retained in the final C-PICASN scale. Reliability indices indicated high internal consistency and stability. Cronbach’s *α* was 0.961 for Factor 1 and 0.950 for Factor 2, with an overall Cronbach’s α of 0.975. Split-half reliability coefficients were 0.949 for Factor 1 and 0.940 for Factor 2, with an overall split-half reliability of 0.952. The test–retest ICC for the total scale was 0.909 (95% CI: 0.831–0.954, *p* < 0.001), indicating excellent temporal stability (Table 3).

**Table 2:** Reliability and item-to-total correlation results of the C-PICASN (*n* = 472).

Items	Item-to-total correlation	Cronbach’s α	Split-half reliability	Test–retest reliability
F1: Basic Competencies as a novice nurse		0.961	0.949	0.891
Item_1	0.816**			
Item_2	0.660**			
Item_3	0.788**			
Item_4	0.732**			
Item_5	0.794**			
Item_6	0.734**			
Item_7	0.694**			
Item_8	0.821**			
Item_9	0.730**			
Item_10	0.851**			
Item_11	0.838**			
Item_12	0.873**			
Item_13	0.778**			
Item_14	0.859**			
Item_15	0.845**			
F2: Relationship-Building Skills within the Healthcare Team		0.950	0.940	0.871
Item_16	0.878**			
Item_17	0.862**			
Item_18	0.651**			
Item_19	0.838**			
Item_20	0.768**			
Item_21	0.788**			
Item_22	0.792**			
Item_23	0.854**			
Item_24	0.569**			
Item_25	0.866**			
Item_26	0.768**			
Item_27	0.820**			
Overall scale		0.975	0.952	0.909

**P < 0.01.

**Table 3:** Test–retest reliability of the C-PICASN (*n* = 30).

Variable	Time 1	Time 2	ICC (95% CI)	*p* value
	Mean ± SD	Mean ± SD		
Factor 1: Basic competencies as a novice nurse	58.4 ± 4.2	58.1 ± 4.5	0.891 (0.802–0.942)	< 0.001
Factor 2: Relationship-building skills within the healthcare team	46.1 ± 3.8	45.9 ± 4.0	0.871 (0.775–0.928)	< 0.001
Overall C-PICASN (total score)	104.5 ± 7.6	104.0 ± 8.1	0.909 (0.831–0.954)	< 0.001

ICC, intraclass correlation coefficient (two-way random effects, absolute agreement). CI, confidence interval. Time 1 and Time 2 were separated by a two-week interval. All *p* values are < 0.001, indicating excellent temporal stability.

### 3.3 Content validity

The C-PICASN demonstrated good content validity. The item-level content validity index (I-CVI) ranged from 0.875 to 1.000, and the scale-level content validity index by average (S-CVI/Ave) was 0.991.

### 3.4 Construct validity

#### 3.4.1 Exploratory factor analysis

Exploratory factor analysis was conducted in a randomly selected subsample of 200 participants. The Kaiser–Meyer–Olkin (KMO) measure was 0.974, and Bartlett’s test of sphericity was significant, with an approximate chi-square value of 12763.070 (*p* < 0.001). Principal component analysis with varimax rotation extracted two factors with eigenvalues greater than 1, which together explained 68.140% of the total variance. All item factor loadings exceeded 0.40. Several items showed cross-loadings (Table 4). For example, the item “It is important for novice nurses to interact with patients intentionally” loaded 0.652 on Factor 1 and 0.488 on Factor 2. Items with cross-loadings were assigned to the factor with the higher loading, and assignments were further supported by clinical judgment and consistency with the theoretical structure. Items with secondary loadings ≥ 0.40 were considered cross-loading. A difference threshold of ≥ 0.20 between primary and secondary loadings was applied for factor assignment. For example, Item 1 loaded 0.652 on F1 and 0.488 on F2 (*Δ* = 0.164), which did not meet the ≥ 0.20 threshold. However, based on theoretical consistency (patient interaction as a basic competency), it was retained under F1. Items with secondary loadings < 0.40 were considered clean. All cross-loadings are reported in Table 4, and final assignments were guided by the higher loading and conceptual alignment with the original scale.

**Table 4:** Factor loadings of the C-PICASN (*n* = 200).

Items	Factors	
	F1	F2
Item_1. It is important for novice nurses to interact with patients intentionally.	**0.652**	0.488
Item_2. It is important for novice nurses to interact with patients with a caring attitude.	**0.702**	
Item_3. It is important for novice nurses to develop a conversation tailored to the patient.	**0.677**	0.418
Item_4. It is important for novice nurses to ask questions to other nurses to ensure patient safety.	**0.792**	
Item_5. It is important for novice nurses to speak from the patient’s point of view.	**0.632**	0.501
Item_6. It is important for novice nurses to use an appropriate professional manner of language.	**0.675**	
Item_7. It is important for novice nurses to dress and groom appropriately as expected of a nurse.	**0.552**	0.423
Item_8. It is important for novice nurses to make great efforts for self-study every day.	**0.794**	
Item_9. It is important for novice nurses to look up things they do not understand on their own.	**0.695**	
Item_10. It is important for novice nurses to ask questions after sorting out what they do not understand.	**0.808**	
Item_11. It is important for novice nurses to understand senior nurses’ advice appropriately.	**0.766**	0.408
Item_12. It is important for novice nurses to greet other staff in a positive manner.	**0.756**	0.471
Item_13. It is important for novice nurses to be honest about their mistakes and not hide them.	**0.798**	
Item_14. It is important for novice nurses to proactively check with senior nurses when they are unsure about something in their work.	**0.783**	0.425
Item_15. It is important for novice nurses to convey words of gratitude to the senior nurses who taught them.	**0.646**	0.551
Item_16. It is important for novice nurses to verbalize what they understand.	0.625	**0.627**
Item_17. It is important for novice nurses to be open to advice from other staff nurses.	0.602	**0.628**
Item_18. It is important for novice nurses to not become emotionally attached to the points raised by nurses in their team.		**0.609**
Item_19. It is important for novice nurses to be express their feelings to other staff nurses within their team during difficult times.	0.565	**0.626**
Item_20. It is important for novice nurses to report at the right time based on the senior nurses’ situation.		**0.794**
Item_21. It is important for novice nurses to adequately report their work progress to senior nurses.		**0.789**
Item_22. It is important for novice nurses to talk with senior nurses without hesitation.	0.491	**0.639**
Item_23. It is important for novice nurses to be specific about the nursing care they want to be supported by senior nurses.	0.602	**0.613**
Item_24. It is important for novice nurses to ask senior nurses to provide care, which they cannot do by themselves.		**0.751**
Item_25. It is important for novice nurses to actively listen to senior nurses’ past experiences for their own growth.	0.541	**0.687**
Item_26. It is important for novice nurses to find reliable seniors in their department.	0.402	**0.703**
Item_27. It is important for novice nurses to proactively support busy nurses within their team.	0.455	**0.721**

EFA summary factor loadings for all 27 items on both factors (F1 and F2). Eigenvalues: Factor 1 = 14.217, Factor 2 = 4.181. % of variance explained: Factor 1: 52.656%, Factor 2: 15.484%, Cumulative variance explained: 68.140%. KMO = 0.974, Bartlett’s test *χ*^2^ = 12763.070 (*p* < 0.001). Cronbach’s α for each factor: F1 = 0.961, F2 = 0.950. Bold values indicate the primary factor loading for each item, which is the higher loading used for item assignment to the corresponding factor. Secondary loadings ≥ 0.40 are reported as cross-loadings.

#### 3.4.2 Confirmatory factor analysis

**Figure 2:**
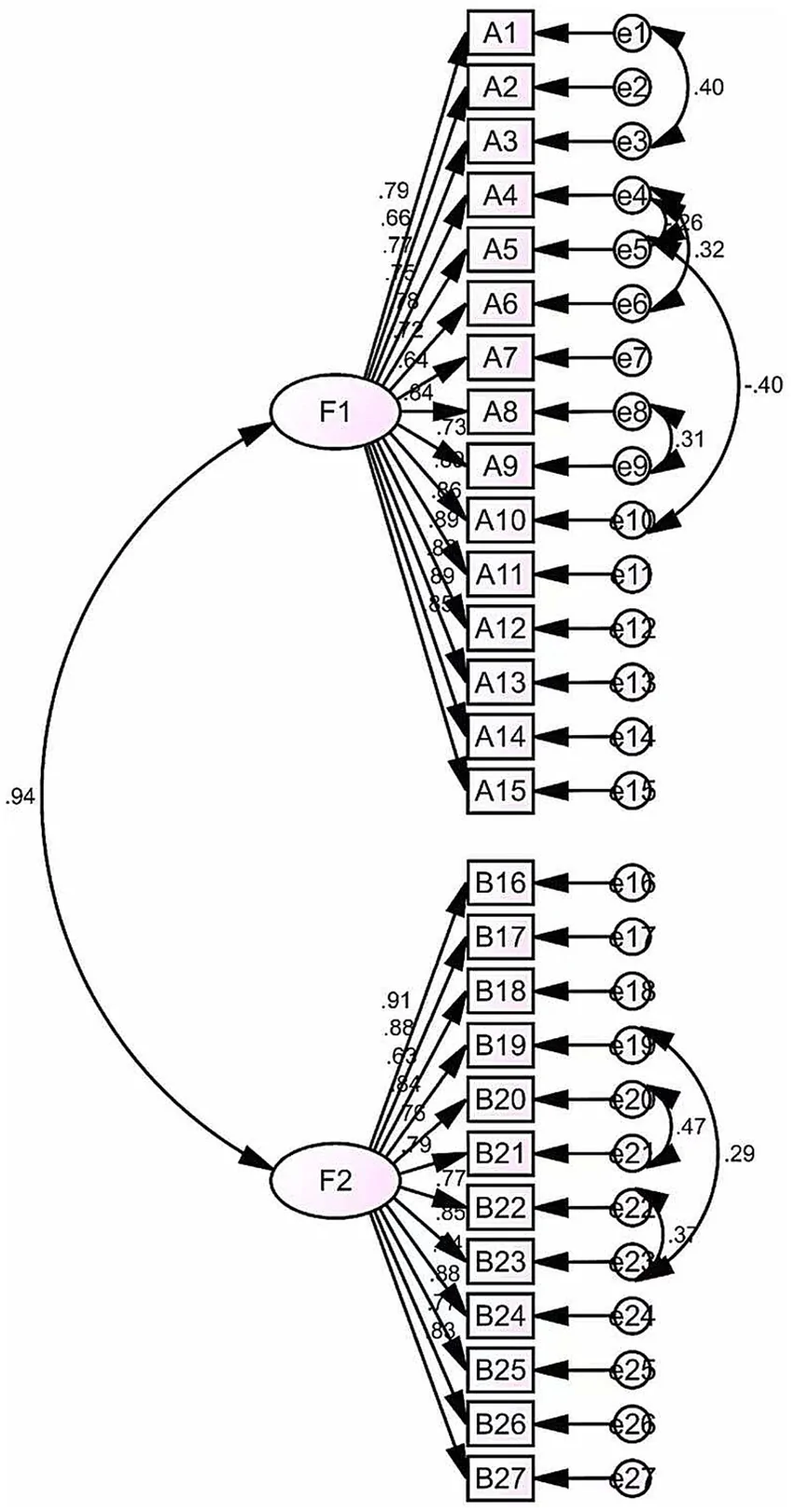
First-order two-factor model of the C-PICASN (*n* = 272). Note: F1: Basic competencies as a novice nurse; F2: Relationship-building skills within the healthcare team.

Confirmatory factor analysis was conducted in the remaining 272 participants to test the first-order two-factor model. The initial model showed suboptimal fit, with χ^2^/df = 5.427, IFI = 0.888, TLI = 0.878, CFI = 0.887, RMSEA = 0.097, and SRMR = 0.046 (Table 5). Based on modification indices (MI > 20) and theoretical justification, the model was refined across eight iterations. The modified model showed improved fit and reached acceptable levels, with χ^2^/df = 4.038, IFI = 0.925, TLI = 0.916, CFI = 0.925, RMSEA = 0.080, and SRMR = 0.040 (Table 5). Multivariate normality was assessed using Mardia’s test (critical ratio = 48.2, *p* < 0.05), indicating non-normal data. Therefore, maximum likelihood estimation with bootstrapping (500 samples) was used. In addition to the reported indices, the GFI was 0.874 and NFI was 0.902 for the modified model, further supporting acceptable fit. Correlated error terms were introduced between items with similar content or wording to account for shared method variance. For example, within Factor 1, e1 and e3 (items related to patient communication), e4 and e5 (items related to ensure patient safety), e4 and e6 (items related to professional language expression), e5 and e10 (items related to asking questions), e8 and e9 (items related to active learning), were correlated, respectively. Within Factor 2, e19 and e23 (items related to seek help from senior nurses), e20 and e21 (items related to reporting to senior nurses), e22 and e23 (items related to senior nurses communication) were correlated. Each modification was reviewed to ensure it did not alter the underlying theoretical structure (i.e., no cross-factor error correlations were added). The final modified model (Figure 2) achieved acceptable fit indices, as shown in Table 5. The standardized factor loadings and the correlation between the two latent factors are presented in Figure 2.

**Table 5:** C-PICASN Confirmatory factor analysis model fitting results (*n* = 272).

**Fit indicators**	**χ** ^**2**^ **/df**	**IFI**	**TLI**	**CFI**	**SRMR**	**RMSEA**	**GFI**	**NFI**
Reference value	2 ~ 5	≥0.90	≥0.90	≥0.90	≤0.05	≤0.08	≥0.90	≥0.90
Initial mode	5.427	0.888	0.878	0.887	0.046	0.097	0.861	0.889
Modified model	4.038	0.925	0.916	0.925	0.040	0.080	0.874	0.902

χ^2^/df-chi-square distribution/degrees of freedom; IFI, Incremental Fit Index; TLI, Tucker-Lewis Index; CFI, Comparative Fit Index; SRMR, Standardized Root Mean Square Residual; RMSEA-Root Mean Square Error of Approximation; GFI, Goodness-of-Fit Index; NFI, Normed Fit Index.

## 4 Discussion

This study translated and culturally adapted the PICASN into Chinese and evaluated the psychometric properties of the Chinese version (C-PICASN) among novice nurses in China. Overall, the findings provide convergent evidence supporting the content validity of the C-PICASN, a two-factor structure consistent with the original conceptualization in this sample, and acceptable reliability across indices., these results indicate that the C-PICASN can be considered for assessing professional interpersonal competency among novice nurses in the Chinese clinical context.

Cross-cultural adaptation is particularly critical for interpersonal competency measures because item meanings may shift across cultural contexts in which hierarchy, communication norms, and role expectations differ (33–35). By following a standardized translation and back-translation approach and incorporating expert review and pilot testing, this study sought to ensure semantic accuracy and conceptual equivalence, thereby reducing the risk of construct drift that can occur when instruments are transferred across languages and cultures. The content validity results further support the appropriateness of the adapted items, with I-CVI values ranging from 0.875 to 1.000 and an S-CVI/Ave of 0.991, exceeding commonly recommended thresholds for adequate content validity (29). These findings suggest that experts considered the C-PICASN items relevant and representative of the construct within the Chinese nursing context, which is important given evidence that hierarchical team structures and power gradients can constrain speaking up and shape interpersonal behaviors in clinical practice.

Construct validity was supported by both exploratory and confirmatory factor analyses (32). In EFA, the KMO value and Bartlett’s test indicated suitability for factor analysis, and the two-factor solution explained a substantial proportion of the total variance. Importantly, this two-domain structure is consistent with the original PICASN framework, which distinguishes basic competencies expected of novice nurses from relationship-building skills within the healthcare team. he presence of cross-loadings for several items is not unexpected in measures of interpersonal constructs, where patient-facing communication and team-based interaction may overlap in real clinical settings. In this study, cross-loading items were retained and assigned to the factor with the higher loading, while also considering clinical interpretability and consistency with the theoretical structure. This approach is consistent with common psychometric practice when primary loadings are adequate and factor assignment is theoretically coherent.

CFA provided further evidence for the two-factor model while also highlighting areas for refinement. The initial CFA model demonstrated suboptimal fit, and iterative model modification improved fit indices to acceptable levels (32). The improved CFI, TLI, and IFI values above 0.90 and the low SRMR are broadly supportive of adequate model fit, whereas the RMSEA of 0.080 lies at the upper bound of commonly used acceptability criteria. Although the modified CFA model achieved acceptable fit indices overall, the RMSEA of 0.080 warrants critical scrutiny. Several factors may contribute to this borderline RMSEA. First, RMSEA is sensitive to model complexity and sample size. Second, the presence of correlated residuals indicates that certain items share variance not fully captured by the latent factors. Thus, we interpret it as suggesting that the model is provisionally acceptable but would benefit from further refinement and replication. A borderline RMSEA may indicate localized model misfit due to content overlap or shared wording among certain items, which can manifest as correlated residuals in CFA. Although modification indices are often used to identify such areas and improve fit, overreliance on data-driven modification can increase the risk of sample-specific overfitting. To mitigate this risk, we adhered to several safeguards: (a) each correlated residual was justified by substantive content overlap rather than statistical criteria alone, (b) no cross-factor error correlations were added, and (c) the theoretical structure of the two-factor model remained intact throughout the modification process. Therefore, the modified model should be interpreted as provisionally supported, with replication in independent samples needed to confirm the stability of the final specification and to strengthen confidence in its generalizability.

Reliability results were consistently strong across indices (36). Item–total correlations were all statistically significant and exceeded the prespecified criterion, indicating satisfactory discrimination and supporting retention of all 27 items. Internal consistency was high for the overall scale and both subscales, and split-half reliability showed similar adequacy. Test–retest reliability over a two-week interval also indicated good temporal stability, which supports the use of the instrument in settings where repeated assessments may be needed. While the high internal consistency (Cronbach’s *α* = 0.975) supports the scale’s reliability, values above 0.95 may suggest item redundancy. This is not unexpected given the conceptual overlap within each of the two domains (e.g., multiple items measuring similar aspects of communication or help-seeking). Future research should explore whether a shortened version of the C-PICASN can maintain measurement precision while reducing respondent burden. Item response theory (IRT) or confirmatory factor analysis with correlated residuals could be used to identify potentially redundant items for deletion.

From an applied perspective, the C-PICASN may help operationalize the assessment of professional interpersonal competency during the vulnerable transition period from student to independent practitioner. Prior work emphasizes that novice nurse retention and adaptation depend not only on technical skill development but also on socialization, mentoring, and supportive team climates that enable feedback, learning, and psychological safety (6, 10–13, 37, 38). Because hierarchical structures may constrain novice nurses’ willingness to speak up, structured assessment of relationship-building skills may also help nursing managers identify needs and tailor interventions such as mentoring programs, communication training, and team-based support strategies that foster constructive interactions and reduce early-career departure (39–43). In this sense, the C-PICASN can support both individual developmental planning and organizational quality improvement initiatives.

Several limitations warrant consideration. First, participants were recruited using convenience sampling within a single province, and a substantial proportion worked in tertiary A-level hospitals, which may limit generalizability to other regions, hospital levels, or specialties. Multicenter studies with more diverse samples are needed to confirm the factor structure and evaluate measurement performance across settings. Second, the cross-sectional design precludes evaluation of predictive validity or responsiveness. Longitudinal research is needed to determine whether C-PICASN scores predict relevant outcomes such as turnover intention, retention, teamwork indicators, and well-being, and whether the instrument is sensitive to changes following training or mentoring interventions. Third, the CFA model required iterative modification to achieve acceptable fit. Although modifications were guided by indices and theoretical considerations, replication in independent samples is essential to reduce the likelihood of overfitting and to establish the stability of the final model. Fourth, test–retest reliability was assessed in a relatively small subsample, and the instrument relies on self-report, which may be influenced by social desirability. Future studies could consider multi-source assessment strategies to strengthen the overall validity evidence. Fifth, while EFA was conducted using principal component analysis (PCA) with varimax rotation, which is common in initial cross-cultural adaptations, PCA is technically a data reduction technique rather than a latent factor extraction method. Future research should confirm the factor structure using principal axis factoring (PAF) or maximum likelihood (ML) factor analysis, which are more appropriate for estimating latent constructs. Sixth, the test–retest reliability was assessed in a relatively small subsample (n = 30), which may limit the precision of ICC estimates. Future studies should include larger retest samples to confirm temporal stability.

To address these limitations and strengthen external validity-, future research should pursue the following directions: First, multicenter validation studies should be conducted across multiple provinces and hospital levels to confirm the factor structure and establish measurement invariance across diverse settings. Second, longitudinal research is needed to evaluate predictive validity. Third, multi-source assessment strategies should be considered. The current instrument relies on self-report, which may be influenced by social desirability bias, particularly in hierarchical cultures where self-promotion or self-criticism may be discouraged. Future studies could incorporate supervisor ratings, peer evaluations, or objective behavioral assessments to provide convergent validity evidence and reduce mono-method bias. Fourth, cross-cultural comparative studies could examine whether the C-PICASN performs equivalently across different cultural contexts within China. Fifth, the scale’s utility in intervention research should be explored. By pursuing these research directions, future studies can establish the C-PICASN’s generalizability, predictive utility, and practical applicability, thereby advancing both the science of interpersonal competency measurement and the practice of supporting novice nurses during their critical transition to independent practice.

## 5 Conclusion

In conclusion, the C-PICASN demonstrated good content validity, a theoretically consistent two-factor structure with acceptable model fit after refinement, and strong reliability among novice nurses in China. Further research should focus on replication across diverse samples, evaluation of external and predictive validity, and potential scale refinement to optimize feasibility for routine clinical use.

## Data Availability

The original contributions presented in the study are included in the article/supplementary material, further inquiries can be directed to the corresponding author/s.
